# The quality in psychiatric care—Addiction outpatient instrument: Psychometric properties and patient views of the quality of care

**DOI:** 10.1002/nop2.861

**Published:** 2021-03-15

**Authors:** Agneta Schröder, Kurt Skårberg, Lars‐Olov Lundqvist

**Affiliations:** ^1^ Faculty of Medicine and Health University Health Care Research Center Örebro University Örebro Sweden; ^2^ Department of Health Science Faculty of Health, Care and Nursing Norwegian University of Science and Technology (NTNU) Gjövik Norway

## Abstract

**Aim:**

The aim of the study was to evaluate the psychometric properties and factor structure of the Quality in Psychiatric Care—Addiction Outpatient (QPC‐AOP) instrument and to describe the experiences with the quality of care among addiction outpatients.

**Design:**

The study has a cross‐sectional design.

**Methods:**

A sample of 244 patients with addiction and psychiatric disorders completed the QPC‐AOP.

**Results:**

Confirmatory factor analysis showed adequate to excellent goodness‐of‐fit indices supporting the 9‐factor structure of the QPC‐AOP. The results thus demonstrate that the concept of quality of care to a large extent is equivalent among outpatients from general psychiatry and from outpatient addiction services. Internal consistency for the full QPC‐AOP was adequate, but poor for some of the separate factors. The patients’ ratings of quality of care were generally high; the highest rating was for Encounter and the lowest for Discharge.

## 1 INTRODUCTION

### 1.1 The outpatient addiction service context

People with substance use disorders are at greater risk for morbidity and mortality (Charlson et al., 2015) and have extensive needs of case management services (Penzenstadler et al., 2017) resulting in high healthcare costs (Darke et al., 2006). In order to meet these needs, and depending on the level of substance abuse, most addiction treatment is organized in inpatient programmes that are intensive, residential treatment programmes designed to treat serious addictions and outpatient programmes that are run part‐time, allowing for less disruption to everyday life for the recovering user. Although there is a discussion on the merits of these programmes (Rossegger et al., 2009; Spinelli & Thyer, 2017), outpatient addiction services can be vital to a person's ability to become drug free as he or she continues working and living his or her life while receiving treatment. However, few studies have evaluated outpatient addiction services from the patients’ perspective.

### 1.2 Measuring quality of care

The quality of the care given is an important factor affecting patient outcome of treatment (Buchanan et al., 2015). In order to ascertain quality, the perceived quality of care needs to be assessed in a reliable and valid way. Assessment of quality of care is usually made from the professionals’ perspective (Baines et al., 2018). However, earlier studies have shown that patients’ and professionals’ perceptions of what defines good quality of care do not always agree (Barbato et al., 2014). Therefore, it has been recognized as necessary to investigate patients’ perceptions of quality of care (Beattie et al., 2015; Taube & Berzina‐Novikova, 2018) as well as including the patient's perspective in measurement development because it contributes to ensuring that the questions asked are relevant and important to the patients (Connell et al., 2018).

However, there are few instruments that have been developed to measure the quality of addiction care (Marsden et al., 2000; Miglietta et al., 2018; Pincus et al., 2016) from the patient's perspective in particular. General instruments have been used instead, which may not be sensitive to the key issues in addiction problems (Marsden et al., 2000). Important key issues identified are, for example, having enough time in care to deal with problems (Bacchus et al., 1999), the interpersonal patient–staff relationship, and planned for and offered aftercare (Lovejoy et al., 1995). A thorough search in databases resulted in only one addiction‐specific instrument where patients have been included in the development of the instrument, namely “The treatment perceptions questionnaire” (TPQ). The TPQ has been developed from a review of existing instruments, research literature and interviews with eight patients and has been designed to measure the treatment satisfaction in addiction treatment programmes (Marsden et al., 2000). There is to the authors’ knowledge no psychometrically tested instrument to measure quality of care in the addiction outpatient care from the patients’ perspective.

### 1.3 Assessing the quality of addiction centre services

Rat et al., (2007) maintained that items based on interviews with patients are more valid than items based on, for example, literature reviews, patient focus groups or professionals. Connell et al., (2018) and Wiering et al., (2017) supported that patients should be involved in the design and wording of the instrument from the beginning as they have unique experience and important information. In addition, instruments measuring quality of care should have a clear definition of the concept (Sitzia & Wood, 1997).

In an endeavour to develop such instruments, the instrument Quality in Psychiatric Care (QPC) was developed. The QPC is a self‐administered instrument measuring different aspects of quality of care. It is based on a definition of quality of care (Schröder et al., 2007, 2010) that has been developed from a phenomenographic interview study with psychiatric in‐ and outpatients (Schröder et al., 2006). The QPC differs substantially from the TPQ instrument in that it has been developed from the patient's perspective and measures different aspects of quality of care.

The aim of the present study was to evaluate the psychometric properties and factor structure of the Quality in Psychiatric Care—Addiction Outpatient (QPC‐AOP) instrument and to describe the experiences with the quality of care among addiction outpatients.

The following research questions were addressed:

‐ Which psychometric properties do the adapted version of the instrument QPC‐AOP have?

‐ How do patients experience the quality of care in psychiatric addiction care?

## 2 MATERIALS AND METHODS

### 2.1 Design

The study has a cross‐sectional design.

### 2.2 Participants and procedure

Data were collected during 8 weeks in April‐May 2014. According to common rules of thumb (Hair et al., 1995), at least 5 respondents per item of the questionnaire are needed, resulting in a recommended sample size of 265. There were 610 patients at the clinics during the data collection period. Questionnaires were returned by 317 patients, but 73 were excluded because they had 30% missing items. The final sample included 244 (144 male and 100 female) addiction patients with aged 18 to 77 (*M* = 39.0, *SD* = 14.1) at centres for outpatient addiction services in two Swedish municipalities, resulting in a response rate of 52%. The centres were staffed by multiprofessional teams and served both urban and rural populations. Patients with different substance abuse problems, such as alcohol, narcotics, anabolic androgenic steroids and pharmaceutical substance abuse problems in addition to one or more psychiatric diagnosis, could be referred to the centres from other caregivers or by self‐referral. All patients who were able to understand and express themselves in Swedish were invited to participate by a contact person at the centres, who also ensured that the participants were cognitively able to answer the questionnaire. Patients were informed about the study both orally and in writing, including information that participation was voluntary and could be terminated at any time. Those who agreed to participate gave their oral consent and were requested to complete the QPC‐AOP questionnaire anonymously and put it in a locked box at the centres. The study was approved by the regional research ethics committee at Uppsala, Sweden (reference number: dnr 2014/040).

### 2.3 Development of the QPC‐AOP instrument

The QPC‐AOP was based on the Quality in Psychiatric Care—Outpatient (QPC‐OP) instrument (Schröder et al., 2011). The QPC‐OP is based on the Quality in Psychiatric Care—Inpatient (QPC‐IP) instrument developed from the patients’ perspective (Schröder et al., 2007, 2010) with certain modifications. The confirmatory factor analysis showed that the factor structure of the QPC‐IP was to a large extent replicated in the QPC‐OP and showed adequate psychometric properties (Schröder et al., 2011). The QPC‐OP consists of 30 items in eight dimensions, which were used verbatim in the QPC‐AOP. Then, 24 items were added to form a tentative addiction‐specific dimension (Table 1). These items were generated from a discussion group with six people who had personal experiences of being a patient in addiction outpatient care. The individuals were recruited from a patients’ addiction association. The discussion group included those with alcohol, narcotics, anabolic androgenic steroids and pharmaceutical substance abuse problems, to form a heterogeneous group that covers patients with various substance abuse problems represented in the addiction outpatient care. They were asked to participate in the discussion group by a contact person. The discussion was led by two of the authors (AS and KS). Notes were taken and the discussion lasted about 2 hr.

**TABLE 1:** Dimensions and items in the QPC‐OP and the QPC‐AOP

QPC‐OP Dimension	QPC‐AOP Dimension^a^
Encounter (6 items)	Encounter (6 items)
Participation–Empowerment (3 items)	Participation–Empowerment (3 items)
Participation–Information (5 items)	Participation–Information (5 items)
Support (4 items)	Support (4 items)
Discharge (3 items)	Discharge (3 items)
Environment (3 items)	Environment (3 items)
Next of kin (2 items)	Next of kin (2 items)
Accessibility (4 items)	Accessibility (4 items)
	Addiction‐specific (23 items)

^a^ Preliminary 53‐item version before psychometric test.

Thereafter, the preliminary QPC‐AOP (54 items in 9 dimensions) was tested for face validity according to Bowling (2005). Six other people with experience of addiction outpatient care recruited from the same patient association were asked to fill in the questionnaire at home and then evaluate it in writing. After one week, they attended a researcher‐led discussion (face‐to‐face) about the questionnaire. The evaluation was carried out using a checklist where each of the 54 items was assessed as either being “Clear and easy to understand,” “Acceptable” or “Unclear and hard to understand.” Each item's importance for representing quality of care in the intended dimension was rated on a 5‐point scale from “very important” to “of little importance.” The questionnaire ratings were discussed by the group. Two of the authors (AS and KS) led the discussions, and notes were taken. The main questions of the discussions were “What's your general impression of the questionnaire?” and “How relevant and useful do you think the specific items are in relation the quality of outpatient addiction care?” The group discussion, which lasted 2 hr, resulted in a rewording of some items and the deletion of one of the 24 addiction‐specific items as it was deemed less relevant to addiction care. All six persons in the second discussion thought the QPC‐AOP was easy to complete within 10–15 min.

The preliminary version of the QPC‐AOP thus included 53 (30 + 23) items concerning quality of addiction outpatient services (Table 1). Each item was related to the statement “I experienced that...” and scored on a 4‐point Likert‐type scale with a rating from 1 (totally disagree)–4 (totally agree). For each item, a “not applicable” alternative was provided. The QPC‐AOP also included background questions about demography and general clinical characteristics.

### 2.4 Data analysis

Descriptive statistical analyses were conducted using SPSS 22 (IBM Corp.). Prior to analysis, imputation was performed by replacing missing data points with the mean of the item in question. The scales were assessed with Cronbach's alpha (Cronbach, 1951), using the 0.70 criterion for adequate homogeneity (Nunnally & Bernstein, 1994). Confirmatory factor analysis (CFA) was used to test the tenability of the a priori proposed factor structure model based on the QPC‐OP. The CFA was performed using LISREL 8.8 (Jöreskog & Sörbom, 1996) with generally weighted least squares estimation on the asymptotic covariance matrices. Polychoric and polyserial correlation matrices were obtained by means of the PRELIS programme (Jöreskog & Sörbom, 1988). The Satorra–Bentler scaled chi‐square (S‐B χ^2^; Satorra et al., 1988) was used to evaluate the adequacy of the model. The S‐B chi‐square permits correct goodness‐of‐fit indices and standard errors for data that are non‐normally distributed. The S‐B chi‐square is, however, sensitive to sample size and even small misspecifications in the model can lead to a significant chi‐square. Consequently, we also evaluated model fit with the comparative fit index (CFI), the standardized root mean square residual (SRMR) and the root mean square of approximation (RMSEA) and its 90% confidence interval (CI). CFI values ≥ 0.95, SRMR values ≤ 0.08 and RMSEA values ≤ 0.06 are viewed as evidence for a well‐fitting model (Hu & Bentler, 1999).

## 3 RESULTS

### 3.1 Descriptive

Most of the patients were Swedish (97%), lived alone (51%) and had no children at home (78%). Most of them (65%) had completed upper secondary school or higher education. About half of them were employed (47%) or looking for employment (29%). About 80% reported one addiction and 16% two addictions, mainly including alcohol (52%), narcotics (43%) or pharmaceuticals (14%). The sample included people with psychiatric disabilities: thirty‐three (14%) reported a neuropsychiatric disorder, sixteen (6%) reported an affective disorder, and nine (4%) reported an anxiety‐related disorder.

### 3.2 Psychometric evaluation of the QPC‐AOP

The confirmatory factor analysis was first performed on the model (Model 1) that represented the 30‐item 8‐factor structure of the QPC‐OP without the addiction outpatient‐specific factor. The CFA showed a significant chi‐square (S‐B χ2 = 467.99, *df* = 377, *p* <.001), a CFI = 1.00, an RMSEA = 0.032 (90% CI = 0.021–0.040) and a SRMR = 0.064, indicating an excellent goodness of fit. Next, since there was no a priori tentative factor structure of the 23 addiction‐specific items, all of them were entered into the model as a specific factor (i.e. a 9th factor). This model (Model 2) was subjected to a CFA, which revealed a significant chi‐square (S‐B χ2 = 2,269.04, *df* = 1,289, *p* <.001), a CFI = 0.96, an RMSEA = 0.056 (90% CI = 0.052–0.060) and a SRMR = 0.030, indicating an adequate goodness of fit. However, several of the 23 items cross‐loaded significantly on at least one of the original factors, thus indicating that the addiction‐specific items did not contribute with any unique aspect of quality of care not already covered by the original items. Therefore, cross‐loading items were excluded from the model in a stepwise manner by successively deleting the item with the highest cross‐loading and re‐evaluating the model until there were no significant cross‐loading items left among the addiction‐specific items. In the final step, four items remained. The CFA of this model, Model 3, showed a significant chi‐square (S‐B χ^2^ = 656.80, *df* = 491, *p* <.001), a CFI = 1.00, an RMSEA = 0.037 (90% CI = 0.029–0.045) and a SRMR = 0.065, indicating an excellent goodness of fit.

As shown in Table 2, the internal consistency of the full 34‐item QPC‐AOP and the Encounter, Participation–Empowerment, Participation–Information, Support, Accessibility and Addiction‐specific factors were adequate. However, the internal consistency of the Discharge, Environment and Next of kin factors did not reach acceptable Cronbach alpha coefficient values.

**TABLE 2:** Summary statistics of confirmatory factor analysis of the QPC‐AOP for patients in psychiatric addiction outpatient care

QPC‐AOP items by dimensions	Loading	Alpha	M	*SD*
Total QPC‐AOP (34 items)		0.96	3.33	0.52
1. Encounter (6 items)		0.93	3.69	0.53
11. Shows empathy	0.91		3.71	0.59
12. Cares if I get angry	0.81		3.63	0.66
15. Respects me	0.94		3.77	0.55
18. Shows understanding	0.93		3.65	0.63
20. Has time to listen	0.95		3.71	0.59
25. Cares about my care	0.88		3.65	0.65
2. Participation–Empowerment (3 items)		0.90	3.28	0.80
1. Influence over my care	0.86		3.10	0.93
5. My view of the right care is respected	0.95		3.35	0.84
6. Take part in decision‐making about my care	0.93		3.37	0.86
3. Participation–Information (5 items)		0.88	3.17	0.71
13. Benefit drawn from earlier experience of treatment	0.75		3.21	0.86
14. Recognize signs of deterioration	0.78		3.02	0.92
27. Given information in a way that can be understood	0.85		3.29	0.79
29. Knowledge about mental troubles	0.86		3.29	0.77
30. Information about treatment alternatives	0.84		3.06	0.97
4. Discharge (3 items)		0.57	2.84	0.59
8. Treatment has helped	0.67		3.25	0.82
17. Help in finding occupation	0.72		2.33	0.69
21. Know where to turn	0.90		2.94	0.88
5. Support (4 items)		0.85	3.35	0.56
19. Stops me from hurting others	0.92		3.32	0.56
22. Stops me from hurting myself	0.68		3.23	0.65
23. Nothing shameful about having mental troubles	0.46		3.48	0.71
24. Shame and guilt must not get in the way	0.57		3.38	0.78
6. Environment (3 items)		0.66	3.48	0.57
2. High level of security at clinic	0.56		3.37	0.73
4. Feel secure with fellow patients	0.80		3.51	0.70
9. Not disturbed by fellow patients	0.85		3.56	0.77
7. Next of kin (2 items)		0.62	3.27	0.69
10. Next of kin invited to take part	0.74		3.07	0.97
28. Respect my next of kin	0.88		3.47	0.61
8. Accessibility (4 items)		0.79	3.25	0.61
3. Easy to meet the contact person	0.78		3.52	0.73
7. Easy to get an appointment	0.84		3.52	0.751
16. Easy to reach the clinic by phone	0.82		3.40	0.773
26. Easy to meet the doctor	0.66		2.57	0.89
9. Addiction‐specific (4 items)		0.92	3.34	0.76
31. Educated about my addiction	0.92		3.36	0.84
32. Help understand risk of relapse	0.92		3.32	0.87
33. Help recognizing signs of relapse risk	0.91		3.12	0.95
34. Staff were knowledgeable about addiction	0.89		3.55	0.73

Note

*N* = 244. QPC‐AOP = Quality of Psychiatric Care—Addiction Outpatient instrument.

### 3.3 Descriptions of quality of addictive outpatient care

Mean and standard deviations of the nine QPC‐AOP dimensions are given in Table 2. Ninety‐two per cent of the participants rated the quality of care as positive (a mean score of 2.5 or higher, which is the centre of the scale). As seen in Table 2, and supported by Bonferroni‐corrected *t* tests, the perceived quality of *Encounter* was significantly greater than the perceived quality of the second ranked dimension, *Environment* (t_(243)_ = 6.69, *p* < .001). The perceived quality of *Environment* was significantly higher than the perceived quality of *Support* (t_(243)_ = 3.826, *p* < .001). There were no significant differences between the perceived quality of *Support*, *Addiction‐specific*, *Participation–Empowerment*, *Next of kin*, *Accessibility* and *Participation*–*Information*. However, *Participation*–*Information* was significantly higher than the lowest ranked dimension, *Discharge* (t_(243)_ = 9.64 *p* < .001).

## 4 DISCUSSION

The QPC‐AOP was designed to capture the quality of addiction outpatient services on the basis of the patients’ own perspective. The results revealed that the factor structure of the QPC‐AOP well fitted the factor structure of the QPC‐OP (Schröder et al., 2011) that it was based upon. Furthermore, the four Addiction‐specific items that were retained fitted the Addiction‐specific factor adequately, thus capturing aspects of addiction services not covered by the other factors in the QPC‐AOP.

Since the QPC‐AOP fitted the a priori factor structure of the QPC‐OP as well as other QPC instruments developed for other psychiatric contexts (Rask et al., 2017; Schröder et al., 2010, 2013), the results indicate that the concept of quality of care among patients has a similar structure regardless of the psychiatric service context.

The internal consistency of the total QPC‐AOP and the dimensions were adequate except in the cases of Discharge, Environment and Next of kin. The low alphas of these three dimensions can probably, in part, be attributed to the low number of items (3, 3 and 2 items, respectively). Further studies are needed to investigate whether this is a replicable phenomenon. These dimensions are thus in need of modification, replacement or addition of further items. Taking this into consideration, the 34 item QPC‐AOP was deemed feasible.

A large number of participants perceived the quality of care as high (i.e. a mean score greater than 2.5, which is the centre of the scale). Earlier studies using other measures of quality of care have shown similarly high ratings of quality of care (Brunt et al., 2019; Jiang et al., 2019; Lally et al., 2013). This is a well‐described issue. In a review study about patients’ satisfaction with mental health treatment, Lebow (1983) found as early as 1983 that less than ten per cent of patients were dissatisfied. We can only speculate about the reasons here, but one explanation may be that patients in psychiatric care have difficulty in criticizing their care (Kondasani and Panda, 2015) in fear of impact on the mental health care they receive (Fernandes et al., 2019). Fernandes et al., (2019) maintain that low participation of patients in the development of items can influence the high ratings. This is, however, not the case in this study as the QPC instrument used was developed from the patients’ perspective and validated among addiction patients as recommended by Kilbourne et al. (2010). In addition, Zieve et al., (2019) and Zimmerman et al., (2017) state that dissatisfaction or low ratings are often associated with dropping out of treatment.

The highest ratings of quality were found in the Encounter dimension, which contains items regarding interpersonal relationships that can be seen as an important aspect in psychiatric care and a core of psychiatric practice (Chambers, 1998) as well in addiction care (Hanpatchalyakul et al., 2012) and may influence the patient's willingness to come back to the care services (Larrabee et al., 2004). Previous studies on quality of psychiatric care show that both out‐ and inpatients put the greatest emphasis on the staff's empathy, on their being interested in, understanding, listening to and respecting patients (Lin et al., 2021; Moreno‐Poyato et al., 2016; Taube and Berzina‐Novikova, 2018) as well as and in other healthcare context (Fang et al., 2019). One qualitative study on how the patients perceived the concept of quality of care confirms the importance of encountering committed and competent staff who understood and confirmed the patients (Schröder et al., 2006). Hanpatchalyakul and colleagues (2012) found the same aspects important in the care and encounter of addiction patients.

The lowest ratings of quality of care were found in the Discharge dimension. This means that the patients are displeased with the help they receive in finding an occupation before they complete the contact with the outpatient addiction care services and do not know where to turn in case they have problems afterwards. The low ratings in the Discharge dimension are consistent with previous findings in outpatient care in Sweden (Schröder et al., 2011). More knowledge is needed about patients’ low ratings of quality of care as patients’ experiences of the care are predictive for patients’ adherence to treatment, quality of life and return to care (Tessier et al., 2017).

The QPC‐AOP was developed based on the QPC‐OP instrument with additional addiction‐specific items. The goodness‐of‐fit result of the analysis including the items from the QPC‐OP was excellent, demonstrating that quality of care aspects in general psychiatric outpatient care were highly relevant for patients in outpatient addiction care too. This suggests that quality of outpatient addiction care is generalizable across different psychiatric contexts. In addition, the QPC‐AOP instrument was developed for outpatient addiction care and intended to be used for improving the quality of care. Additional instruments for psychiatric addiction inpatient care and care staff are needed in order to compare the different perspectives on rating quality of care. In addition, translations of the QPC‐AOP to other languages are important for cross‐cultural examinations which can be used in further nursing‐related research in the topic area.

### 4.1 Limitations

The goodness of fit of the QPC‐AOP was excellent; however, there were below adequate levels of internal consistency of some individual dimensions. Given that these dimensions consisted of few items (two to three), lower internal consistency is to be expected. The sample size was at the lower end of the sample size criterion and since this was the first time the addiction‐specific items were evaluated, a new and larger sample is needed to confirm the adequacy of these items. It was impossible to analyse the dropouts because of incomplete “missing” patient registrations at the participating clinics. Although the main objective of the study was psychometric evaluation, it should be noted that the cross‐sectional design precludes conclusions about cause and effect.

### 4.2 Implications for practice

The findings in this study imply several clinical recommendations that invites for reflection in the improvement of the addiction outpatient services and nursing education. Firstly, the knowledge about patients’ ratings of low quality of care regarding the dimension Discharge can be used in the improvement of the quality of care and nursing education for the purpose to improve the information to the patients so that they know where to ask for help if they need care after the contact was completed. Secondly, QPC‐AOP can be routinely used as a self‐rating instrument for the purpose of improving psychiatric addiction outpatient care and can constitute a part in a quality system. Thirdly, QPC‐AOP can be used as a tool to motivate care staff, especially nurses, to continually and systematically improve the care as the QPC‐AOP is a simple, inexpensive and quick way to evaluate quality of care.

## 5 CONCLUSIONS

The QPC‐AOP is a psychometrically adequate instrument for evaluating patients’ experiences of the quality of addiction services. Based on a patient perspective, the QPC‐AOP will contribute to the improvement of addiction services and the development of theory in this area. Clinical recommendations for nursing are to use validated instruments, such QPC‐AOP, for follow up the results and to increase the professional knowledge about patients’ views of quality of care.

## CONFLICT OF INTEREST

The authors declare no conflicts of interest with respect to the research, authorship and/or publication of this article. This research received no specific grant from any funding agency in the public, commercial or not‐for‐profit sectors.

## AUTHORS’ CONTRIBUTION

All authors have contributed significantly and are in agreement with the content of the manuscript.

## Data Availability

Data available on request from the authors. The data that support the findings of this study are available from the corresponding author upon reasonable request.
